# Western Equine Encephalitis Virus in Blood Donors during Outbreak, Argentina, 2023–2024

**DOI:** 10.3201/eid3208.260078

**Published:** 2026-08

**Authors:** Sebastián Blanco, María C. Frutos, Kevin Brenot, Alejandra González Bustamante, Luis H. Carrizo, Sandra V. Gallego

**Affiliations:** Universidad Católica de Córdoba, Córdoba, Argentina (S. Blanco); Universidad Nacional de Córdoba, Córdoba (S. Blanco, M.C. Frutos, K. Brenot, S.V. Gallego); Fundación Banco Central de Sangre, Córdoba (S. Blanco, A. González Bustamante, L.H. Carrizo, S.V. Gallego); Consejo Nacional de Investigaciones Científicas y Técnicas, Buenos Aires, Argentina (M.C. Frutos, S.V. Gallego)

**Keywords:** western equine encephalitis virus, viruses, transfusion-transmitted infections, arboviruses, blood bank, blood safety, asymptomatic viremia, molecular surveillance, public health, vector-borne infections, Argentina

## Abstract

Western equine encephalitis virus (WEEV) reemerged in Argentina during 2023–2024, raising public health concerns and posing a potential threat to blood transfusion safety. We assessed the effect of WEEV circulation on the blood supply by implementing responsive, community-informed interventions within a regional hemovigilance system, including epidemiologic mapping, enhanced donor selection criteria, and alphavirus-specific nucleic acid testing. Among 637 donors residing in outbreak-affected areas, we detected 1 asymptomatic viremic donor. Phylogenetic analysis showed that the viral sequence clustered within the contemporary South American outbreak lineage, consistent with ongoing regional transmission. Those findings demonstrate silent WEEV circulation among blood donors and highlight the critical role of donor surveillance as a sentinel system for emerging arboviruses. Hemovigilance provides valuable insights into viral circulation in the broader population, underscoring the need to integrate molecular surveillance into routine blood bank practices to strengthen transfusion safety and public health preparedness.

Arthropodborne viruses (arboviruses) are a diverse group of pathogens transmitted to vertebrate hosts through the bite of infected hematophagous arthropods, such as mosquitoes, ticks, and sand flies, and are recognized as an important public health concern. Representative arboviruses, including West Nile virus (WNV), dengue virus, Zika virus, and chikungunya virus (CHIKV), are an ongoing concern for blood safety because of their potential for transfusion-transmitted infections (TTIs) (*1*–*4*). Moreover, the possible effect of mosquitoborne virus outbreaks may affect both the safety and availability of the blood supply (*5*–*8*). A substantial proportion of arbovirus infections might be asymptomatic or subclinical, although that varies by virus. Because those infections can still produce transient viremia, infected persons could donate blood without being aware of their infectious status (*9*). Therefore, several countries have implemented donor deferral policies or nucleic acid testing (NAT) to mitigate transfusion risk, including routine WNV NAT screening in the United States and Canada and a combination of 28-day donor deferral and NAT screening strategies across countries in Europe, such as the United Kingdom, Germany, Italy, and Greece (*6*–*8*).

In that context, the reemergence of western equine encephalitis virus (WEEV) in Latin America during 2023–2024 represents an important public health concern (*10*–*12*). In Argentina, several ecologic and epidemiologic factors may have contributed to the reemergence of the virus. One factor potentially associated with this outbreak was a public policy decision made in 2016 by the Servicio Nacional de Sanidad y Calidad Agroalimentaria (SENASA), which modified the mandatory annual vaccination strategy against eastern equine encephalitis virus (EEEV) and WEEV (*13*). The decision made immunization voluntary, resulting in a substantial decline in vaccination coverage among the equine population. The reemergence led to a major outbreak in humans; the last human case before then was recorded in 1996.

Equine encephalomyelitis viruses are maintained in nature through enzootic transmission cycles involving birds, small mammals, and mosquito vectors, which complicates surveillance (*11*–*13*). Under certain ecologic conditions, spillover to horses may occur, leading to outbreaks in horses and, occasionally, in humans (*10*–*12*). Like other alphaviruses, WEEV can induce viremia in humans during the early stages of infection, which is asymptomatic in most cases. Consequently, subclinical infections are highly frequent among infected persons. Estimated symptomatic-to-asymptomatic ratio is ≈1:58 in children <4 years of age and 1:1,150 in adults (*14*), which is one of the main reasons it is so difficult to determine the true effect of WEEV circulation in the general population.

During epidemiologic week 48 of 2023 through week 26 of 2024, SENASA confirmed 1,529 equine outbreaks across 17 provinces; no new outbreaks were detected after week 16 of 2024. In humans, 572 suspected cases were reported across 21 provinces; 107 were confirmed, 21 were classified as probable, and 115 were discarded. The last confirmed cases were reported in week 15 of 2024. Confirmed cases occurred across all age groups (median age 58 years, range 4 months–81 years); 60% of cases were in persons 50–69 years of age, 86% were in male patients, and 14% were in female patients. A total of 12 deaths were reported among confirmed cases (*14*). Among the fatal cases, 8 had exposure to rural or semirural settings and 7 had underlying conditions. The deceased persons were 30–74 years of age; 10 were male and 2 were female. Confirmed cases of encephalitis have also been reported by clinicians (*15*,*16*).

When extrapolating the number of reported symptomatic cases to the estimated total number of infections on the basis of symptomatic-to-asymptomatic ratios, the true magnitude of the outbreak is likely higher than officially reported. Given the high proportion of asymptomatic infections and the absence of laboratory screening tools or specific donor deferral criteria for WEEV, a potential risk to blood safety exists during outbreaks. Moreover, confirmed human cases in Argentina were concentrated among persons within the age range eligible for blood donation. Because asymptomatic persons might experience transient viremia early in the infection, they could contribute to unrecognized viral circulation. Consequently, the risk for undetected viremic donations and potential transfusion transmission remains uncertain. Hemovigilance could therefore provide valuable evidence for assessing transfusion safety along with insights into the broader circulation of the virus in the general population.

In this study we aimed to evaluate the potential effect of WEEV circulation on transfusion safety. We assessed the likelihood of silent viremia among blood donors during the 2023–2024 outbreak in Argentina and explored the value of implementing hemovigilance strategies as a sentinel system for epidemiologic surveillance capable of generating evidence to inform and guide public health decision-making.

## Methods

Within the framework of WEEV case notification in Córdoba, Argentina, the Hemovigilance System of the Fundación Banco Central de Sangre (FBCS) developed intervention strategies tailored to the regional epidemiologic context and the operational characteristics of the blood bank. Those strategies, aimed at safeguarding transfusion safety, included the creation of epidemiologic maps to geolocate infections, the establishment of blood donor selection criteria, and the selection of plasma samples from donors residing in areas affected by the virus for the specific detection of WEEV RNA by NAT.

We implemented the strategies and interventions at the FBCS, a reference blood bank in Córdoba, the second most populous province in Argentina. The FBCS coordinates and centralizes the activities of multiple blood transfusion services operating across the province’s 165,321-km^2^ territory. Approximately 50% of all blood units collected throughout Córdoba undergo serologic and molecular pretransfusion screening at FBCS. Consequently, the donor population represented in this blood bank is not confined to a specific locality but covers a broad geographic area in the central region of the country. 

### Epidemiologic Maps

As part of the outbreak response, real-time epidemiologic maps were developed to track suspected WEEV circulation and identify emerging hotspots across the province. FBCS obtains its blood supply through external collection campaigns organized throughout the province. Medical and technical teams travel daily to different locations to recruit eligible donors and collect blood units. Because official epidemiologic reports did not specify the localities where cases were detected, FBCS began collaborating with community promoters and local blood drives organizers to gather information on suspected cases in their areas. In addition, whenever FBCS teams traveled to a suspicious region, potential donors were asked during the predonation information and screening process whether they were aware of any probable cases in their communities. The information collected through those channels enabled the creation of maps highlighting hotspots of suspected areas. Hotspots were defined as geographic areas with repeated reports of suspected WEEV circulation based on field-collected epidemiologic information. Those maps made it possible to visualize the progressive spread of the outbreak across extensive areas of the province.

### WEEV-Specific Risk Assessment Criteria for Donor Selection

In addition to the standard criteria for blood donor selection, we implemented additional criteria to ensure the quality and safety of the collected blood. The personnel responsible for donor eligibility interviews were instructed to ask targeted questions designed to identify whether potential blood donors may have been exposed to WEEV. The questions were designed to identify potential exposure scenarios, including suspected or probable equine encephalitis cases in animals and humans within the donor’s household, neighborhood, workplace, or other regularly visited settings.

Personnel interviewing donors considered the following aspects during the selection process of blood and blood component donors and recorded all collected information in the donation form. First, for residence or travel history, they asked whether donors currently lived in affected areas or had visited or vacationed in such areas within the previous 15 days. Second, for local disease occurrence, they asked about suspected or probable cases of equine encephalitis in animals or humans near the donor’s area of residence. Third, for human exposure, they asked whether there were suspected cases among household members, coworkers, or other persons with whom the donor frequently interacts (e.g., relatives or friends). Fourth, for animal exposure, they asked whether there were suspected cases among animals owned by the donor or others with whom the donor has frequent contact (e.g., at work, or at the homes of relatives or friends they regularly visit). Finally, personnel considered deferral criteria; donors who resided with or frequently visited persons or animals suspected of infection, or who had had the disease within the past 15–30 days, could not donate for >1 month.

### Reinforcement of Postdonation Symptom Reporting

Donors were strongly reminded to promptly report any signs or symptoms that occur after blood donation. To raise awareness among donors, donation staff informed them that a person may be infected and capable of transmitting an infection before clinical symptoms become apparent. They emphasized that timely reporting of any postdonation signs or symptoms can prevent the use of collected blood units, thereby reducing the risk for transfusion-transmitted infections.

### Sample Selection

We randomly and retrospectively selected plasma samples from persons identified as residents of WEEV-affected regions from the FBCS Hemovigilance Program serolibrary, where sample aliquots are cryopreserved for hemovigilance studies. Donations included in the study were collected during the outbreak period, beginning with the first reported cases in late January 2024 and extending through the period of vector circulation in April 2024 (epidemiologic weeks 3–14).

### Nucleic Acid Test

We performed viral RNA extraction from plasma samples using the High Pure Viral Nucleic Acid kit (Roche). We performed reverse transcription to complementary DNA using random hexamer primers and the M-MLV reverse transcription enzyme (Promega). We detected the WEEV genome using a previously described nested PCR targeting members of the *Alphavirus* genus (*17*) that amplifies a 195-bp fragment of the conserved nonstructural protein 4 region of alphaviruses. We purified amplification products using the commercial QIAquick Gel Extraction Kit (QIAGEN). We sequenced the purified DNA in both directions (Table). 

**Table:** Genome sequences used for phylogenetic analysis in study of western equine encephalitis virus in blood donors during outbreak, Argentina, 2023–2024*

Accession no.	Isolate	Location	Host	Year	Lineage
GQ287640	McMillan	Canada	*Homo sapiens*	1941	A
KJ554965	California	United States	*Equus caballus*	1930	A
KT844544	Y62–33	Russia	*Aedes cinereus*	1962	A
KT844545	CU71-CPA	Cuba	NA	1971	A
MN477208	Fleming	United States	*Homo sapiens*	1938	A
GQ287644	BFS-2005	United States	*Culex tarsalis*	1974	B1
KJ554966	BFS932	United States	*Culex tarsalis*	1946	B1
KJ554968	BFS1703	United States	*Culex tarsalis*	1953	B1
KJ554969	E1416	United States	*Zonotrichia leucophrys*	1961	B1
KJ554973	75V9291	United States	*Culex tarsalis*	1975	B2
KJ554977	PV02808A	United States	*Culicidae*	1990	B2
KJ554980	93A38	United States	*Culicidae*	1992	B2
KJ554984	CNTR34	United States	*Culex tarsalis*	1993	B2
KJ554985	Lake43	United States	*Culex tarsalis*	1994	B2
KT844548	SUYA140	United States	*Culex tarsalis*	1993	B2
KT844550	Kern87	United States	*Culex tarsalis*	1996	B2
KU978771	97–5067	United States	*Meleagris gallopavo*	1996	B2
KU978772	98–2435	United States	*Dromaius novaehollandiae*	1997	B2
NC_003908	71V1658	United States	NA	1971	B2
GQ287641	Imperial	United States	*Culex tarsalis*	2005	B3
GQ287643	Montana-64	United States	*Equus caballus*	1967	B3
GQ287647	85–452NM	United States	*Culex tarsalis*	1985	B3
KJ554970	S8–122	United States	*Hesperosciurus griseus*	1968	B3
KJ554971	BT-235	United States	*Gopherus berlandieri*	1971	B3
KJ554972	BFS3060	United States	*Culex tarsalis*	1971	B3
KJ554978	IMPR441	United States	*Culex tarsalis*	1992	B3
KJ554983	93A79	United States	*Culicidae*	1993	B3
KJ554986	PV72102	United States	*Culicidae*	1997	B3
KJ554987	PV012357A	United States	*Culicidae*	2001	B3
KJ554988	R02PV002957B	United States	*Culicidae*	2002	B3
KJ554991	R0PV00384A	United States	*Culicidae*	2005	B3
KT844547	CHLV31	United States	*Culex tarsalis*	1985	B3
KT844543	CBA87	Argentina: Cordoba province	*Equus ferus*	1958	C
PP544260	EQ1090	Brazil	*Equus caballus*	2023	C
PP669618	EQ1122	Brazil	*Equus caballus*	2023	C
PP669617	EQ237	Brazil	*Equus caballus*	2024	C
PP620641	DILAVE070	Uruguay	*Equus caballus*	2023	C
PP620642	DILAVE236	Uruguay	*Equus caballus*	2023	C
PP620643	DILAVE218	Uruguay	*Equus caballus*	2023	C
PP620644	DILAVE158	Uruguay	*Equus caballus*	2023	C
PP620645	DILAVE198	Uruguay	*Equus caballus*	2024	C
PP620646	DILAVE255	Uruguay	*Equus caballus*	2024	C
KT844541	TR25717	Guyana	*Equus ferus*	1959	None
KT844542	Ar_Enc_MV	Argentina: Buenos Aires province	*Equus ferus*	1993	None
NC_075015	AG80–646	Argentina: Chaco province	*Culex*	1980	None
PP669617	EQ237–2024	Brazil	*Equus ferus*	2023	C
PP544260	EQ1090–2023	Brazil	*Equus ferus*	2023	C
PV641588	ARG_CBA_354546–1	Argentina: Cordoba province	*Homo sapiens*	2024	C

*All sequences obtained from GenBank. NA, not available.

### Phylogenetic Analysis

We performed multiple sequence alignment using MAFFT version 7.450 (https://mafft.cbrc.jp/alignment/software). We performed phylogenetic analyses using the maximum-likelihood method in W-IQ-TREE software (*18*) and the best-fit nucleotide substitution model selected by ModelFinder (*19*). We evaluated robustness of the phylogenetic grouping by the SH-like approximate-likelihood ratio test using 1,000 replicates (*20*) estimated with ModelFinder in IQ-TREE. We considered a clade with an SH-aLRT >80% well-supported. We visualized phylogenetic trees with FigTree version 1.4.4 (https://tree.bio.ed.ac.uk/software/figtree).

## Results

After the report of a human case of WEEV infection in Córdoba (January 26, 2024), through epidemiologic maps, we identified areas of suspected WEEV circulation (hotspots). We implemented interventions in the affected areas from February 5, 2024, through the first week of April 2024 (epidemiologic weeks 6–14). The staff of the mobile blood collection teams scheduled to operate in those areas, as well as the personnel at fixed blood collection sites located within the identified hotspots, were instructed to conduct donor selection in accordance with the procedures we described. In addition, we used updated donor information materials to reinforce the importance of reporting postdonation reaction.

During the intervention period, a total of 4,048 persons (56% male, 44% female; mean age 38 years, interquartile range [IQR] 28–52 years) donated blood at fixed donation centers and external collection sites. Of those donors, 637 (49% male, 51% female; mean age 38 years, IQR 32–47 years) resided in areas identified by our mapping system as having suspected WEEV circulation. Most locations identified as hotspots were in rural areas where many residents, including altruistic repeat blood donors, live in close contact with horses and maintain close domestic contact with them. Eligible donors identified as potentially exposed to WEEV on the basis of our additional selection criteria were temporarily deferred from donation. For example, applying our selection criteria enabled us to identify persons otherwise eligible to donate blood as close contacts of someone who had died from WEEV infection.

We processed all samples from donors identified as living in hotspots by alphavirus NAT; we identified 1 viremic donor. We deposited the sequence obtained from PCR product into GenBank (accession no. PV641588). The phylogenetic analysis revealed that sequence clustered within the lineage associated with WEEV isolates detected during the 2023–2024 outbreak in South America (Figure). Specifically, we observed the highest genetic similarity to sequences reported from Argentina and Uruguay during that period, suggesting a close epidemiologic relationship with the viral strains responsible for that regional outbreak. In the tree, the sequence, PV641588 ARG CBA 354546 (Argentina, 2024), clustered within the recently proposed C sublineage (*11*); we found no evidence of introduction from North America or other continents in our analysis. Because the sequence is genetically very close to circulating equine and mosquito isolates, our findings are consistent with a human infection occurring in the context of the equine–mosquito enzootic/epizootic cycle characteristic of the C sublineage in South America.

**Figure:**
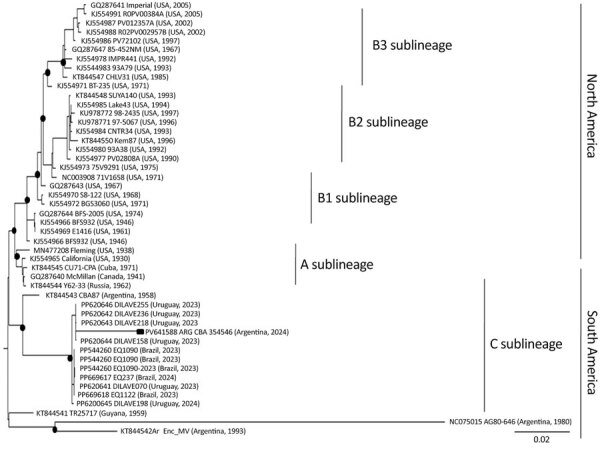
Phylogenetic analysis from study of western equine encephalitis virus (WEEV) in blood donors during outbreak, Argentina, 2023–2024. We generated a maximum-likelihood phylogenetic tree of a partial nucleotide sequence nonstructural protein 4 (201 bp) of a WEEV isolate from Argentina (black square) compared with other sequences from GenBank (accession numbers shown). We edited the tree using FigTree version 1.4.4 (https://tree.bio.ed.ac.uk/software/figtree). We selected the best-fit nucleotide substitution model general time-reversible substitution model with estimated base frequencies and gamma-distribution rate with 4 discrete categories according to the Bayesian Information Criterion. Black dots at nodes show the highest support (>80%) by SH-like approximate-likelihood ratio test. WEEV strains from the 2023–2024 outbreak group closely with other reported strains from Uruguay and Brazil (C sublineage). We defined WEEV lineages in accordance with previous research (*22*). Scale bar indicates number of nucleotide substitutions per site.

## Discussion

Among emerging viruses with public health impact that threaten transfusion safety, arboviruses are a major concern because of their ability to cause asymptomatic viremia, their rapid geographic expansion, and the absence of systematic pretransfusion screening tests. In response to the WEEV outbreak in Argentina, we identified the need to implement specific interventions to assess potential risks to transfusion safety.

Collaboration with community promoters proved valuable during the WEEV outbreak, providing localized epidemiologic insights not captured in official public health reports. Information gathered from community promoters and local blood drive organizers enabled real-time situational awareness and enabled the FBCS to identify areas of suspected viral circulation. In addition, information reported by potential donors during predonation screening, particularly regarding suspected or probable cases within their communities, contributed valuable on-the-ground data that helped build a more complete picture of WEEV circulation across the province. That combined flow of community-based and donor-reported data contributed to shaping adaptive measures, guiding risk assessment, and refining donor selection criteria. Overall, our findings suggest that community engagement can enhance transfusion safety during outbreaks and underscore the importance of flexible, locally informed strategies to protect the blood supply in the face of emerging infectious threats.

During WEEV reemergence, the rapid adaptation of donor selection criteria by our blood banks was important to safeguard the blood supply. Standard eligibility assessments may not adequately capture evolving exposure risks. Incorporating targeted questions enabled a more rigorous evaluation of donor risk during those critical periods. Of note, the development of the questions was strengthened by information provided by community promoters, whose knowledge of local idiosyncrasies, customs, and daily practices offered valuable insights into potential exposures. Thus, integrating context-specific data into standard donor eligibility screening supported the implementation of appropriate deferral measures to prevent TTIs. Moreover, the 30-day donor deferral period reflected a risk-based approach tailored to a complex epidemiologic context, characterized by the concurrent circulation of WEEV and a large dengue outbreak in 2024. Although longer deferral periods could further reduce the theoretical risk for transfusion transmission, extending deferral beyond 30 days would likely compromise the availability of critical blood components in a system already under strain. Those findings underscore the importance of adaptive context-informed donor selection strategies to maintain both transfusion safety and blood supply resilience during emerging infectious threats

Our study revealed through NAT that an asymptomatic viremic donor might pose a risk for TTI despite implementation of targeted questions to minimize transfusion risk. That finding is consistent with the high frequency of asymptomatic infections associated with WEEV and highlights an inherent limitation of symptom-based and exposure-based donor screening; namely, a residual risk. We found no evidence of inaccurate disclosure during donor history assessment; rather, the most plausible explanation is that at the time of donation the donor was in the asymptomatic viremic phase, which cannot be identified through standard predonation screening. Therefore, in viral infection scenarios involving a high proportion of asymptomatic persons, molecular screening should complement targeted donor selection strategies to meaningfully reduce residual TTI risk. In a previous hemovigilance study (*21*), we proposed a cost-efficient molecular screening strategy for arboviruses in blood donors based on a NAT assay configured with universal degenerate primer pairs targeting conserved genomic regions, which could detect a broad range of viruses in a single reaction. That generic screening platform could enable a rapid response to emerging threats such as WEEV. Beyond transfusion safety, molecular screening of blood donors also can function as a valuable surveillance tool for uncovering silent viral circulation. Because routine surveillance systems rely on clinical reporting of symptomatic cases, asymptomatic infections often remain undetected. In that context, monitoring an asymptomatic population such as blood donors can fill an important surveillance gap by helping to identify otherwise unrecognized transmission and complementing traditional surveillance systems, thereby supporting the detection of emerging threats and the identification of transmission patterns and hotspots (*9*,*21*).

Molecular screening of blood donors not only reveals WEEV circulation among eligible donors but also enables characterization of human-derived viral strains. The isolate we identified, PV641588, did not form a distinct lineage; instead, it clustered with contemporary South America strains associated with the mosquito–equine transmission cycle (2023–2024) (*10*–*12*), supporting shared transmission dynamics across hosts. Phylogenetic analysis showed that PV641588 belongs to the recently described C sublineage (*11*), which has circulated in South America for decades. The clustering of recent isolates from Argentina, Uruguay, and Brazil is consistent with a possible regional intensification of viral circulation. The placement of PV641588 within that group supports the hypothesis that the human infection occurred in the context of active enzootic transmission, rather than representing a divergent lineage.

Our findings suggest that responsive, community-informed strategies can strengthen transfusion safety while helping to maintain the continuity and resilience of the blood supply during periods of epidemiologic uncertainty. They also reinforce the value of molecular screening in blood banks as a surveillance tool capable of detecting silent viral circulation that traditional clinical reporting systems may miss. Together, our results highlight the role of blood donors as sentinels for emerging arboviral threats and support integrating molecular surveillance into routine blood bank practices to strengthen public health preparedness.

## References

[1] Pealer LN, Marfin AA, Petersen LR, Lanciotti RS, Page PL, Stramer SL, et al.; West Nile Virus Transmission Investigation Team. Transmission of West Nile virus through blood transfusion in the United States in 2002. N Engl J Med. 2003;349:1236–45. 10.1056/NEJMoa030969 14500806

[2] Giménez-Richarte Á, Ortiz de Salazar MI, Giménez-Richarte MP, Collado M, Fernández PL, Clavijo C, et al. Transfusion-transmitted arboviruses: update and systematic review. PLoS Negl Trop Dis. 2022;16:e0010843. 10.1371/journal.pntd.0010843 36201547 PMC9578600

[3] Gould CV, Free RJ, Bhatnagar J, Soto RA, Royer TL, Maley WR, et al.; Yellow Fever Vaccine Virus Transplant and Transfusion Investigation Team. Transmission of yellow fever vaccine virus through blood transfusion and organ transplantation in the USA in 2021: report of an investigation. Lancet Microbe. 2023;4:e711–21. 10.1016/S2666-5247(23)00170-2 37544313 PMC11089990

[4] Almeida FJ, Pacheco JT, Farias CGA, de Matos SF, de Morais CO, Guerra GG, et al. Dengue: a hidden threat in blood transfusions amidst Brazil’s largest outbreak? Lancet Infect Dis. 2025;25:e10. 10.1016/S1473-3099(24)00795-3 39622264

[5] Teo D, Ng LC, Lam S. Is dengue a threat to the blood supply? Transfus Med. 2009;19:66–77. 10.1111/j.1365-3148.2009.00916.x 19392949 PMC2713854

[6] Giménez-Richarte Á, de Salazar MO, Arbona C, Giménez-Richarte MP, Collado M, Fernández PL, et al. Prevalence of chikungunya, dengue and Zika viruses in blood donors: a systematic literature review and meta-analysis. Blood Transfus. 2022;20:267–80. 10.2450/2021.0106-21 34694219 PMC9256504

[7] Frank C, Schmidt-Chanasit J, Ziegler U, Lachmann R, Preußel K, Offergeld R. West Nile virus in Germany: an emerging infection and its relevance for transfusion safety. Transfus Med Hemother. 2022;49:192–204. 10.1159/000525167 36159956 PMC9421668

[8] Moreira-Soto A, Postigo-Hidalgo I, Tabares X, Roell Y, Fischer C, Gotuzzo E, et al. Transfusion-transmitted infections: risks and mitigation strategies for Oropouche virus and other emerging arboviruses in Latin America and the Caribbean. Lancet Reg Health Am. 2025;46:101089. 10.1016/j.lana.2025.101089 40458696 PMC12127558

[9] Blanco S, Marín ÁL, Frutos MC, Barahona NY, Rivarola ME, Carrizo LH, et al. Haemovigilance survey and screening strategy for arthropod-borne viruses in blood donors from Argentina. J Med Virol. 2024;96:e29476. 10.1002/jmv.29476 38373210

[10] Tomás G, Marandino A, Rodríguez S, Wallau GL, Dezordi FZ, de Oliveira ALS, et al. Diagnosis and genomic characterization of the largest western equine encephalitis virus outbreak in Uruguay during 2023–2024. Npj Viruses. 2024;2:70. 10.1038/s44298-024-00078-6 40295695 PMC11721398

[11] Campos AS, Franco AC, Godinho FM, Huff R, Candido DS, da Cruz Cardoso J, et al. Molecular epidemiology of western equine encephalitis virus, South America, 2023–2024. Emerg Infect Dis. 2024;30:1834–40. 10.3201/eid3009.240530 39173662 PMC11346983

[12] Vissani MA, Alamos F, Tordoya MS, Minatel L, Schammas JM, Dus Santos MJ, et al. Outbreak of western equine encephalitis virus infection associated with neurological disease in horses following a nearly 40-year intermission period in Argentina. Viruses. 2024;16:1594. 10.3390/v16101594 39459927 PMC11512283

[13] Servicio Nacional de Sanidad y Calidad Agroalimentaria (SENASA). Resolución 521/2016. Boletín Oficial de la República Argentina. 2016 Sep 12 [cited 2026 May 8]. https://www.boletinoficial.gob.ar/detalleAviso/primera/150852/20160915

[14] Ministerio de Salud de la Nación. Boletín Epidemiológico Nacional no. 700. 2024 [cited 2026 May 8]. https://www.argentina.gob.ar/sites/default/files/2024/04/ben_700_se15_vf.pdf

[15] González Pannia P, De Lillo L, Roldán M, Miño L, Pruscino F, Farias E, et al. Western equine encephalitis: a pediatric case report. Arch Argent Pediatr. 2025;123:e202410392. 10.5546/aap.2024-10392.eng 39207945

[16] Campassi ML, Cuitiño M, Dorregaray F, Litardo Banegas DM, Repetto FG, Fauez C. Western equine encephalitis: brainstem involvement and clinical manifestations. Medicina (B Aires). 2024;84:1240–5. 39666418

[17] Sánchez-Seco MP, Rosario D, Quiroz E, Guzmán G, Tenorio A. A generic nested-RT-PCR followed by sequencing for detection and identification of members of the alphavirus genus. J Virol Methods. 2001;95:153–61. 10.1016/S0166-0934(01)00306-8 11377722

[18] Trifinopoulos J, Nguyen LT, von Haeseler A, Minh BQ. W-IQ-TREE: a fast online phylogenetic tool for maximum likelihood analysis. Nucleic Acids Res. 2016;44(W1):W232–5. 10.1093/nar/gkw256 27084950 PMC4987875

[19] Kalyaanamoorthy S, Minh BQ, Wong TKF, von Haeseler A, Jermiin LS. ModelFinder: fast model selection for accurate phylogenetic estimates. Nat Methods. 2017;14:587–9. 10.1038/nmeth.4285 28481363 PMC5453245

[20] Guindon S, Dufayard JF, Lefort V, Anisimova M, Hordijk W, Gascuel O. New algorithms and methods to estimate maximum-likelihood phylogenies: assessing the performance of PhyML 3.0. Syst Biol. 2010;59:307–21. 10.1093/sysbio/syq010 20525638

[21] Blanco S, Frutos MC, Spinsanti L, Gallego SV. Silent St. Louis encephalitis virus circulation evidence by a haemovigilance survey in a centralized blood bank. J Public Health (Oxf). 2025;47:e56–8. 10.1093/pubmed/fdae303 39611757

[22] Bergren NA, Haller S, Rossi SL, Seymour RL, Huang J, Miller AL, et al. “Submergence” of Western equine encephalitis virus: evidence of positive selection argues against genetic drift and fitness reductions. PLoS Pathog. 2020;16:e1008102. 10.1371/journal.ppat.1008102 32027727 PMC7029877

